# Response of Root Growth and Development to Nitrogen and Potassium Deficiency as well as microRNA-Mediated Mechanism in Peanut (*Arachis hypogaea* L.)

**DOI:** 10.3389/fpls.2021.695234

**Published:** 2021-06-11

**Authors:** Lijie Li, Qian Li, Kyle E. Davis, Caitlin Patterson, Sando Oo, Wanying Liu, Jia Liu, Guo Wang, Julia Elise Fontana, Thomas Elliott Thornburg, Isaac Seth Pratt, Fei Li, Zhiyong Zhang, Yanzhong Zhou, Xiaoping Pan, Baohong Zhang

**Affiliations:** ^1^Henan Collaborative Innovation Center of Modern Biological Breeding, Henan Institute of Science and Technology, Xinxiang, China; ^2^Department of Biology, East Carolina University, Greenville, NC, United States; ^3^Elizabeth City State University, Elizabeth City, NC, United States; ^4^College of Life Sciences, Anhui Normal University, Wuhu, China; ^5^Peanut Research Institute, Luohe Academy of Agricultural Sciences, Luohe, China

**Keywords:** peanut, nitrogen deficiency, potassium deficiency, microRNA, gene expression

## Abstract

The mechanism of miRNA-mediated root growth and development in response to nutrient deficiency in peanut (*Arachis hypogaea* L.) is still unclear. In the present study, we found that both nitrogen (N) and potassium (K) deficiency resulted in a significant reduction in plant growth, as indicated by the significantly decreased dry weight of both shoot and root tissues under N or K deficiency. Both N and K deficiency significantly reduced the root length, root surface area, root volume, root vitality, and weakened root respiration, as indicated by the reduced O_2_ consuming rate. N deficiency significantly decreased primary root length and lateral root number, which might be associated with the upregulation of miR160, miR167, miR393, and miR396, and the downregulation of AFB3 and GRF. The primary and lateral root responses to K deficiency were opposite to that of the N deficiency condition. The upregulated miR156, miR390, NAC4, ARF2, and AFB3, and the downregulated miR160, miR164, miR393, and SPL10 may have contributed to the growth of primary roots and lateral roots under K deficiency. Overall, roots responded differently to the N or K deficiency stresses in peanuts, potentially due to the miRNA-mediated pathway and mechanism.

## Introduction

Cultivated peanut (*Arachis hypogaea* L.) is one of the three major legume grain and oilseed crops in China, with a planting area of over 5.0 × 10^6^ ha and annual pod yields of more than 1.6 × 10^7^ t in recent years, which plays a significant role in ensuring nutritional food security in China (Zhang et al., 2020). However, since the soil has been frequently over-exploited in modern agricultural practices, nitrogen (N) and potassium (K) have been the important limiting factors. Therefore, they were often frequently supplemented in the form of fertilizers (Kuicheski et al., 2015).

There is a large amount of N fertilizer usage all over the world. Still, N fertilizer’s recovery rate or efficiency is relatively low in arable land, accounting for only 25–50% of the application amount (Sharma and Bali, 2018; Omara et al., 2019). K deficiency is very common. For example, in China, more than one-third of cultivated soil is in the state of K deficiency (soil available K content is 50–70 mg/kg) or serious K deficiency (soil available K content is less than 50 mg/kg; Wang and Wu, 2009). Therefore, it is of great significance for optimizing nutrient management, particularly the supply of N and K in balance with crop demand and for the genetic improvement of peanut to explore the response mechanism of N deficiency and K deficiency in peanut.

Since N is involved in synthesizing important components such as proteins, nucleic acids, phospholipids, chlorophyll, enzymes, vitamins, alkaloids, and certain growth hormones in plants, it is also called the element of life (Hawkesford et al., 2012; Kant, 2018). The common symptoms of N deficiency include leaf yellowing, cell division slowing down, and enzyme activity decreasing and then leading to plant growth retardation (Salvagiotti et al., 2008). Compared with N, K does not directly participate in synthesizing any chemical substances in plants, but it is the most abundant cation in plants (Salvagiotti et al., 2008). It is widely distributed in various tissues and organs of plants, accounting for about 2–10% of the dry weight of plants (Leigh and Wyn Jones, 1984). K plays a very important role in maintaining cytoplasmic charge balance, regulating enzymatic reaction, promoting protein synthesis, and maintaining cell osmotic pressure and other physiological activities (Leigh and Wyn Jones, 1984). At the same time, K also participates in a series of physiological and biochemical processes, such as stomatal movement, cell signal transduction, cell elongation, and phloem material transport (Sustr et al., 2019). Proper K application is conducive to regulating nutrient transport and distribution in peanuts, promoting nodule formation, improving nodule nitrogen fixation capacity (Wang et al., 2013), regulating carbon and N metabolism, and improving peanut yield, quality, and resistance (Helmy and Ramadan, 2014; Almeida et al., 2015; Chakraborty et al., 2016).

The root is the first plant organ to sense and absorb nutrients, and their morphological response to different growth conditions varies greatly (Gruber et al., 2013). Plant roots show a high degree of plasticity for changes in the availability of nutrient resources, and the degree of this plasticity depends on the plant species in many cases (Sun et al., 2017; Sustr et al., 2019). Apart from the role as a nutrient element, nitrate or ammonium (the main form of N that can be taken by the roots) acting as a signal regulate many physiological processes, including the development of root system architecture (RSA; Sun et al., 2017; Kant, 2018) by regulating cell division and expansion (Luo et al., 2020). The growth and development of RSA depend on K supply that affected root differentiation and elongation (Sustr et al., 2019). Sufficient cytoplasmic K level is required for protein synthesis and enzyme activity in root cells to maintain cytoplasmic pH (Walker et al., 1998) and protein anion charge (Maathuis and Sanders, 1996). In addition, the cells in the elongation region need expansion pressure, which is established by osmotically active substances (including K; Pritchard, 1994; Dolan and Davies, 2004). In the mature zone of roots, root hairs grow through K flux (Guilhem et al., 2003; Zhao et al., 2016). In addition, the RSA and root hair coverage rate in plants changed adaptively, thus improving K uptake under K limitation (Guilhem et al., 2003; Zhao et al., 2016).

Plants have evolved several physiological and molecular adaptive responses to cope with nutrient deficiency stresses (Kuicheski et al., 2015; Nguyen et al., 2015). microRNAs (miRNAs) are short (21–24 nucleotide) RNAs, which can bind to RNA-induced silencing complex (RISC), recognize its target mRNAs, and regulate the expression of the targeted genes through transcriptional cleavage and translation inhibition (Zhang et al., 2006). If miRNA and mRNA are completely complementary, miRNA cleaves the phosphodiester bond between two nucleotides of the target mRNA sequence (generally between the 10th and 11th nucleotides of miRNA), that is to guide the specific cleavage of mRNA; if not, it will cause translation inhibition (Zhang et al., 2006; Sunkar et al., 2012). Studies have shown that miRNAs can regulate the quantity and spatiotemporal accumulation of target mRNAs and thus play a key regulatory role in plant stress response (Sunkar et al., 2012; Zhang, 2015), including in nutrient deficiency stress (Kuicheski et al., 2015; Nguyen et al., 2015).

Studies have shown that nutrient availability would change the expression of miRNAs and their target mRNAs and then affect the growth and development of plants (Kuicheski et al., 2015). Furthermore, different crops respond differently to nutritional deficiencies (Kuicheski et al., 2015; Fontana et al., 2020; Thornburg et al., 2020). However, the mechanism of miRNAs related to root growth and development in response to nutrient deficiency in peanuts is still unclear. In this work, we systematically explored the response of root development and morphology to N deficiency and K deficiency and the potential miRNA-mediated mechanism during these signs of progress. The observation of the expression of the miRNAs and their targeted mRNAs in peanut plants exposed to N deficiency and K deficiency could reveal the mechanisms of the plant suffering from nutrient deficiency stress and help find candidate genes to develop important tools for plant nutrition breeding.

## Materials and Methods

### Plant Growth Conditions, Nutrient Stress Treatments, and Sampling

The peanut (*Arachis hypogaea* L. cv. Yuanza 9,102) seeds of the same size, fully mature, and free of diseases and insect pests were selected, soaked in 10% H_2_O_2_ solution for 5 min washed with deionized water five to six times. The seeds were wrapped in absorbent paper, soaked in a plastic bucket containing 2 L saturated CaSO_4_, and placed in the artificial climate chamber (constant temperature 28°C, blue light irradiation) for germination. When the root length of peanut seedlings was about 10 cm (4 days), the seedlings with uniform appearance were selected and planted in plastic pots containing 5 L of modified Hoagland nutrient solution (pH 6.3 ± 0.1): (1) normal N (NN) nutrient solution; (2) low N (LN) nutrient solution; (3) normal K (NK) nutrient solution; (4) low K (LK) nutrient solution. The nutrient solution group of NN consists of: 2.5 mM Ca(NO_3_)_2_, 2.5 mM KCl, 2 mM NaCl, 1 mM MgSO_4_, 0.5 mM KH_2_PO_4_, 0.1 mM EDTA-FeNa, 2 × 10^−2^ mM H_3_BO_3_, 1 × 10^−3^ mM ZnSO_4_, 1 × 10^−3^ mM MnSO_4_ 2 × 10^−4^ mM·L^−1^ CuSO_4_, and 5 × 10^−6^ mM (NH_4_)_6_Mo_7_O_24_. Compared with the nutrient solution group of NN, the 2.5 mM Ca(NO_3_)_2_ was replaced by 0.05 mM Ca(NO_3_)_2_ and 2.45 mM CaCl_2_ in the nutrient solution group of LN. The nutrient solution group of NK consists of: 2.5 mM Ca(NO_3_)_2_, 2.5 mM KCl, 2 mM NaCl, 1 mM MgSO_4_, 0.5 mM NH_4_H_2_PO_4_, 0.1 mM EDTA-FeNa, 2 × 10^−2^ mM H_3_BO_3_, 1 × 10^−3^ mM ZnSO_4_, 1 × 10^−3^ mM MnSO_4_, 2 × 10^−4^ mM·L^−1^ CuSO_4_, and 5 × 10^−6^ mM (NH_4_)_6_Mo_7_O_24_. Compared with the nutrient solution group of NN, the 0.5 mM KH_2_PO_4_ was replaced by 0.5 mM NH_4_H_2_PO_4_ in the nutrient solution group of NK. Compared with the nutrient solution group of NK, the 2.5 mM KCl was replaced by 0.02 mM KCl and 2.48 mM NaCl in the nutrient solution group of LK. The nutrient solution was changed every 3 days to avoid nutrient depletion. The light source of the growth chamber was a biological sodium lamp. The light/dark period was 14/10 h, and the day and night temperature were 30 ± 2°C/25 ± 2°C. A certain amount of deionized water was added to the culture container every day to supplement the water consumed by transpiration. The air pump was used for ventilation for 24 h. Four and eight days after the nutrient stress treatments, the plants were collected to determine the dry and fresh weight and conduct root morphological analysis; the roots were collected to measure root respiration and root vigor. In addition, the root samples from each treatment were harvested and frozen in liquid nitrogen immediately and then stored at −80°C before RNA extraction.

### Plant Growth Determination

Five peanut plants were randomly selected, and the substrate on the surface of the root and dust on the surface of leaves were washed with deionized water. After the surface water was wiped off, the peanut plants were divided into two parts: the aboveground part and the root part, and the fresh weight of each part was weighed. First, the plant height was measured with a ruler. Next, all leaves were scanned by a desktop scanner (EPSON Perfection V700 Photo), and pictures were taken. Then, WinRHIZO was used to analyze the leaf area (cm^2^). Finally, the samples were dried at 105°C for 30 min and then to a constant weight at 80°C. Then, the dry weights of aboveground and roots were weighed, respectively.

### Root Morphological Analysis

The primary root length was measured with a ruler. Then, counts were taken for the lateral root number. After that, the whole root was scanned with a desktop scanner (EPSON Perfection V700 Photo), and root pictures were taken. Finally, WinRHIZO was used to analyze the length (cm), surface area (cm^2^), volume (cm^3^), and diameter (mm) of the root samples.

### Determination of Root Vitality

Root vitality was determined according to triphenyltetrazolium chloride (TTC) staining method. The roots of five seedlings from each treatment were weighed and cut into a small fragment that was ~1 cm in length. Then the root was soaked in 20 ml of 0.6% TTC solution prepared with 0.1 mol/L phosphate buffer (pH = 7.4). After 24 h, the buffer solution was poured out, and the roots were washed with deionized water three times. Then the roots were soaked in 20 ml 95% ethanol and a water bath at 85°C for 10 min to extract water-insoluble triphenylmethyl hydrazone (TTF). The absorbance of the extract was measured at 485 nm, and the root vitality was expressed as OD g^−1^ FW.

### Root Respiration Rate Measurement

The roots of five seedlings from each treatment were weighed and put into the incubator chamber of the Clark Chlorolab2 system. Then, 2 ml saturated CaSO_4_ solution was added, and the oxygen (O_2_) consumption was measured after 10 min. The O_2_ consumption rate was calculated and represented by the root respiration rate, expressed as nmol min g^−1^ FW.

### RNA Extraction and Gene Expression Analysis

About 200 mg of root samples of peanut seedlings on the 4th and 8th days of N deficiency, K deficiency, and the control treatments were used to extract total RNAs. According to our previous reports, RNA extraction and isolation were performed as outlined in the manufacturer’s protocols of the MirVana miRNA Isolation Kit (Candar-Çakir et al., 2016; Fontana et al., 2020; Yu et al., 2020). The quality of the extracted RNAs was firstly tested by Nanodrop ND-1000 (Nanodrop Technologies, Wilmington, DE, United States). One percentage agarose gel electrophoresis was used, and the quality and concentrations of RNAs were detected by micro ultraviolet spectrophotometer. Then the extracted RNA was stored at −80°C for reverse transcription.

According to the procedures of the manufacturer of TianGen miRcute Plus miRNA First Strand cDNA first Synthesis Kit (Beijing, China), the RNA samples were reverse transcribed into cDNAs with miRNA gene-specific primers and pol(T) primers of all protein-coding genes. Then the synthesized cDNA was stored at −20°C used for further analysis.

To investigate the potential mechanism of miRNA-mediated plant response to N deficiency and K deficiency, we targeted miRNAs related to plant root development and/or the dynamic balance of nutrients in other species (Nguyen et al., 2015; Li and Zhang, 2016; Zhang and Unver, 2018; Song et al., 2019). After considering all these criteria, a total of 20 miRNAs were selected for this study. They were miR156, miR160, miR162, miR164, miR165, miR166, miR167, miR169, miR171, miR172, miR319, miR390, miR393, miR395, miR399, miR396, miR778, miR827, miR847, and miR857. The expression of seven targets genes (*NAC4*, *AFB3*, *ARF1*, *ARF2*, *bHLH74*, *SPL10*, and *GRF*) found in the literature was also analyzed. The expression of miRNAs and their target genes were analyzed with the gene-specific forward and reverse primers according to the Tiangen miRcute Plus miRNA qPCR detection kit (Beijing, China). Each gene and miRNA expression analysis included three biological replicates and three technical replicates. The expression value was standardized with reference gene EF1A. The ΔΔCT method was used to calculate the fold change of the tested miRNAs and their target genes.

### Statistical Analysis

The data of each morphological and physiological index was calculated by five biological repeats, and gene expression was obtained by three biological repeats. Statistical software SPSS version 19 was employed for ANOVA and followed by the least significant difference (LSD) test. A value of *p* less than 0.05 and 0.01 was considered a significant difference and extremely significant difference and marked as * and ** in the graphs.

## Results

### Effects of N and K Deficiency on the Growth and Development of Peanut Seedlings

**Figure 1:**
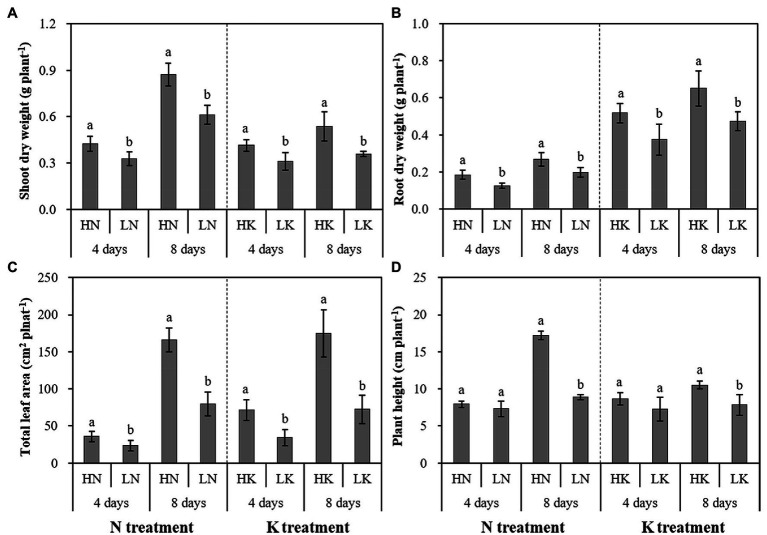
The effect of N deficiency and K deficiency on the shoot dry weight **(A)**, root dry weight **(B)**, total leaf area **(C)**, and plant height **(D)** of peanut seedlings, respectively. The data are the means ± SDs (*n* = 5). The different letters on the bars indicate significant differences between the different N (or K) treatments and the control treatment on the same day according to the LSD test (*p* = 0.05). N, nitrogen; K, potassium; and LSD, least significant difference.

As shown in Figure 1, the dry weight of shoot and root and leaf areas significantly decreased under both N and K deficiency on the 4th and 8th days. In addition, the plant height was significantly decreased under N and K deficiency on the 8th day. Compared with the seedlings cultured in the normal nutrient solutions, the dry weight of shoot, root, and total plant of the seedlings cultured in N deficiency nutrient solutions on the 8th day decreased by 29.67, 25.73, and 18.60%, respectively, and that of the seedlings cultured in K deficiency nutrient solutions on 8th day decreased by 32.97, 27.15, and 14.29%, respectively. In addition, the leaf area and the plant height of the seedlings cultured in N deficiency nutrient solutions on the 8th day decreased by 51.79 and 48.37%, respectively, and that of the seedlings cultured in K deficiency nutrient solutions on the 8th day decreased by 58.51 and 25.54%, respectively.

### Effects of N and K Deficiency on Root Development of Peanut Seedlings

**Figure 2:**
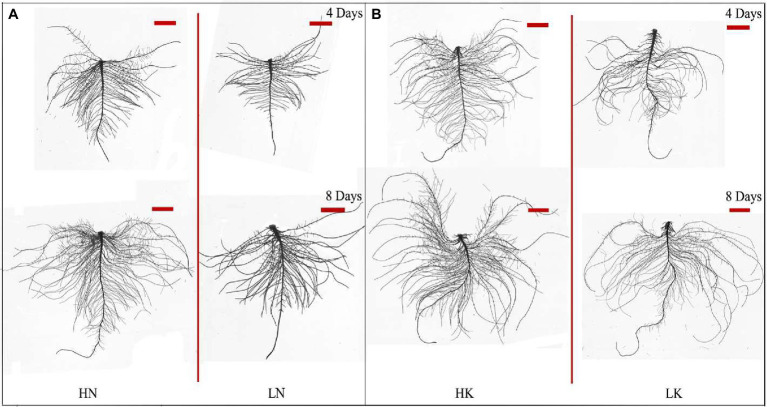
Root morphology difference between high N (HN) group and low N (LN) group **(A)** and the difference between high K (HK) group and low K (LK) group **(B)** after 4 and 8 days of treatments. N, nitrogen and K, potassium. The original size of the target bar is 0.2 cm high and 3.5 cm wide.

**Figure 3:**
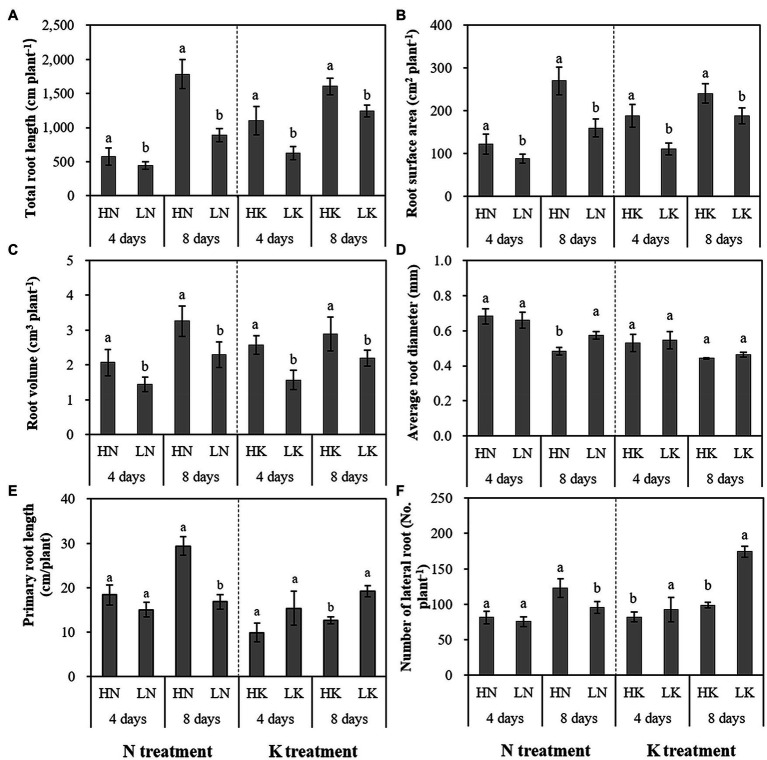
The effect of N deficiency and K deficiency on the total root length **(A)**, root surface area **(B)**, root volume **(C)**, average root diameter **(D)**, primary root length **(E)**, and the number of lateral roots **(F)** of peanut seedlings, respectively. The data are the means ± SDs (*n* = 5). The different letters on the bars indicate significant differences between the different N (or K) treatments and the control treatment on the same day according to the LSD test (*p* = 0.05). N, nitrogen; K, potassium; and LSD, least significant difference.

N and K deficiency had significant effects on root development, indicated not only by the root biomass but also the root length, size, and number of branches (Figure 2). As shown in Figure 3, compared with the seedlings cultured in the normal nutrient solutions, the total root volume and total root surface area as well as the total root length of the seedlings, cultured in N deficiency nutrient solutions on the 8th day, decreased by 50.29, 40.78, and 29.50%, respectively, and that of the seedlings cultured in K deficiency nutrient solutions on 8th day decreased by 19.06, 21.57, and 24.42%, respectively. On the other hand, compared with the seedlings cultured in the normal nutrient solutions, the average root diameter had no significant change under K deficiency conditions. At the same time, it increased significantly under 8 days of low N treatment.

N and K deficiency effects on the primary root length and lateral root number of peanut seedlings were opposite. N deficiency significantly reduced the primary root length of the seedlings on both the 4th and 8th days of N deficiency treatment. In addition, it decreased the lateral root number of the seedlings on the 8th day of N deficiency treatment. In contrast, K deficiency significantly promoted the growth of the primary root and increased the lateral root number of the seedlings on the 8th day. Compared with the seedlings cultured in the normal nutrient solutions, the primary root length and the lateral root number of the seedlings cultured in N deficiency nutrient solutions on the 8th day decreased by 44.89 and 22.49%, respectively, and that of the seedlings cultured in K deficiency nutrient solutions on 8th day increased by 59.04 and 71.46%, respectively.

### N and K Deficiency Affected Root Vigor and Root Respiration in Peanut Seedlings

**Figure 4:**
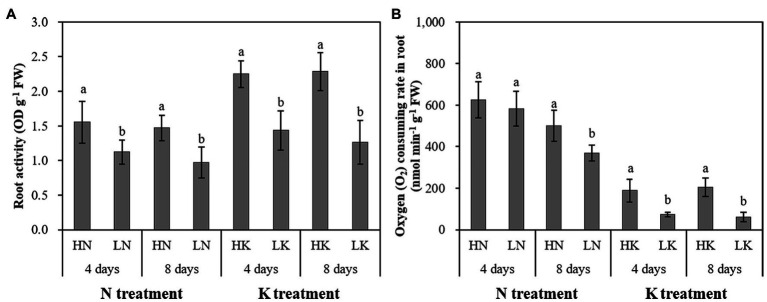
The effect of N deficiency and K deficiency on the root activity **(A)** and oxygen (O_2_) consuming rate in the root **(B)** of peanut seedlings, respectively. The data are the means ± SDs (*n* = 5). The different letters on the bars indicate significant differences between the different N (or K) treatments and the control treatment on the same day according to the LSD test (*p* = 0.05). N, nitrogen; K, potassium; and LSD, least significant difference.

N and K deficiency significantly impacted the growth and development of roots and significantly affected the health of roots, which was manifested by changes in root vigor and respiration (Figure 4). On both the 4th and 8th days of treatment, the root vigor was significantly changed under both N deficiency and K deficiency treatments, and with the extension of the stress time, the root vigor showed a gradual decline trend. Compared with the seedlings cultured in the normal nutrient solutions, the root vigor of the seedlings cultured in N deficiency nutrient solutions and the seedlings cultured in K deficiency nutrient solutions on the 8th day of treatment decreased by 33.84 and 44.64%, respectively. On the 4th day of treatment, there was no significant difference between the root respiration rate of seedlings grown in N deficient nutrient solution and that of seedlings grown in a normal nutrient solution. However, with the extension of stress time, the root respiration kept decreasing. The difference of the root respiration between N deficiency treatment and the controls reached a significant level on the 8th day of treatment. The root respiration of the seedlings cultured in K deficiency nutrient solutions showed a gradual decline trend, and the difference of the root respiration between the seedlings grown in K deficient nutrient solution and the seedlings grown in normal nutrient solution reaches a significant level on both 4th and 8th days of K treatment.

### N Deficiency and K Deficiency Changed the Expression of miRNAs and Their Targeted mRNAs

**Figure 5:**
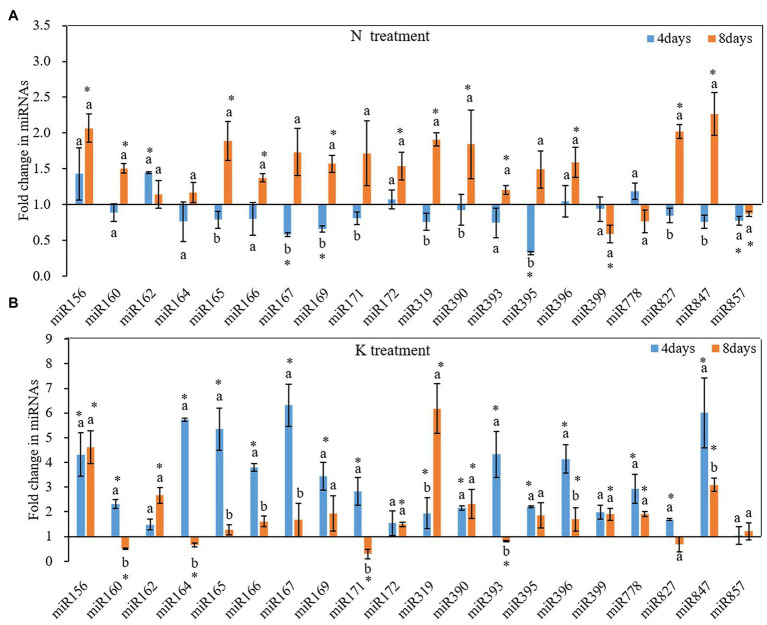
Fold change of microRNAs (miRNA) expression between N deficiency and the controls **(A)** and between K deficiency and the controls **(B)** in the root of peanut seedlings, respectively. Error bars represent SD (*n* = 3). The different letters on the bars indicate significant differences in the fold change of miRNA target expression between 4 and 8 days of treatment according to the LSD test (*p* = 0.05). A value of *p* less than 0.05 was considered as a significant difference between N deficiency and the controls **(A)**, and between K+ deficiency and the controls **(B)**, which marked as ∗ in the graphs. N, nitrogen; K, potassium; and LSD, least significant difference.

N and K deficiency changed the expression of miRNAs and their targeted mRNAs (Figure 5). However, the response of different miRNAs to N deficiency and K deficiency was different. On the 4th day of N deficiency treatment, except miR156, miR162, miR172, miR396, miR778, the expression of other miRNAs was inhibited, and the inhibition of the expression of miR167, miR169, miR395, miR399, and miR857 reached a significant level compared with that in the controls (Figure 5A). In contrast, on the 8th day of N deficiency treatment, except miR399, miR778, and miR857, the expression of the other tested miRNAs was induced compared with that in the controls, and the induction of the expression of miR156, miR160, miR165, miR166, miR167, miR169, miR171, miR172, miR319, miR390, miR393, miR396, miR827, and miR847 reached a significant level compared with that in the controls. On the 4th day of K deficiency treatment, all the 20 miRNAs were induced (Figure 5B). Among them, miR167 was upregulated the most by 6.32-fold, and the expression of miR156, miR164, miR165, miR166, miR169, miR393, miR396, and miR847 were also upregulated by more than threefold change. On the 8th day of treatment, although the expression of most miRNAs (except for miR160, miR164, miR171, miR393, and miR827) under K deficiency treatments were still higher than that in the control treatment, the value of fold changes was much lower than that determined on 4th day of treatments. On the 8th day of treatments, the expression of miR160, miR164, miR171, and miR393 under K deficiency treatment was significantly lower than that under the controls.

**Figure 6:**
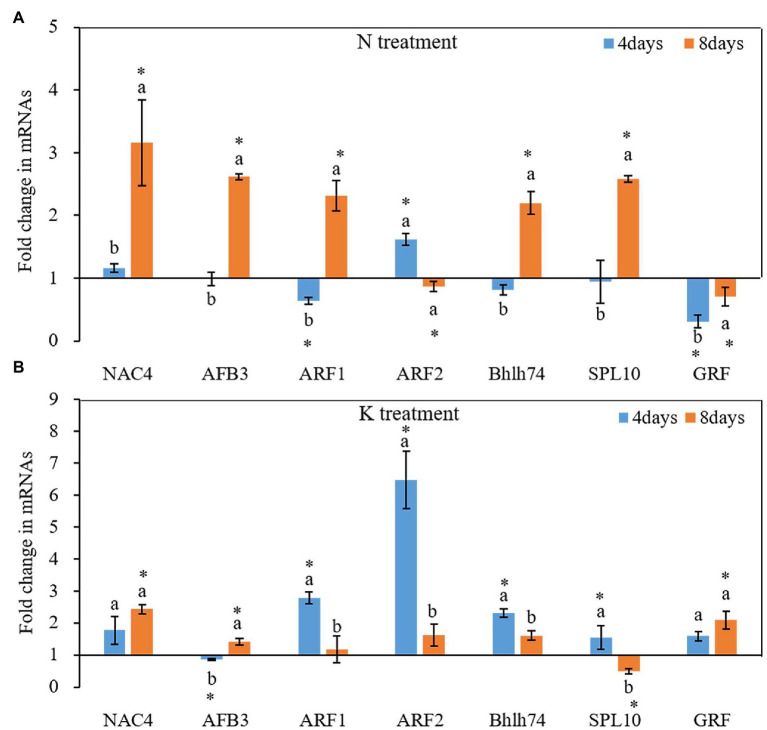
Fold change of miRNA target gene expression between N deficiency and the controls **(A)** and between K deficiency and the controls **(B)** in the root of peanut seedlings, respectively. The different letters on the bars indicate significant differences in the fold change of miRNA target expression between 4 and 8 days of treatment according to the LSD test (*p* = 0.05). A value of *p* less than 0.05 was considered as a significant difference between N (or K) deficiency and the controls, which marked as ∗ in the graphs. N, nitrogen; K, potassium; and LSD, least significant difference.

N and K deficiency also changed the expression of protein-coding genes (Figure 6). On the 4th day of treatments, the expression of all tested seven miRNA-targeted genes, except NAC4 and ARF2, was inhibited under N deficiency treatment, and the induction of the expression of ARF1 and GRF reached a significant level compared with that in the controls (Figure 6A). In contrast, after 8 days of treatments, the total of potential miRNA targets, except the ARF2 and GRF gene, was induced by N deficiency treatment. Compared with the control, among these genes, NAC4 had the highest expression under N deficiency treatment, and the fold change value reached 3.46. On both the 4th and 8th days of K deficiency treatment, all tested seven potential miRNA targets, except AFB3 gene on 4th day of treatments and SPL10 on 8th day of treatments, were up-expressed in the root of seedlings grown in K deficient nutrient solution than that in normal nutrient solution (Figure 6B). ARF2 was the most upregulated among the seven target genes, and the fold changes up to 6.47-fold on the 4th day of K deficiency condition. Although the expression of most potential miRNA targets in the root of seedlings grown in K deficient nutrient solution was still higher than that of the control treatment on the 8th day of treatment, the fold change was much smaller than that determined on the 4th day of treatment. ARF1, ARF2, bHLH74 and SPL10 transcription factor gene expression levels were decreased by 57.75, 75.04, 30.29, and 68.25%, respectively, on the 8th day of treatments compared with that on 4th day of treatments under K deficiency treatments.

### The Relationship Between miRNAs and Their Targeted mRNAs Under N and K Deficiency Treatments

**Figure 7:**
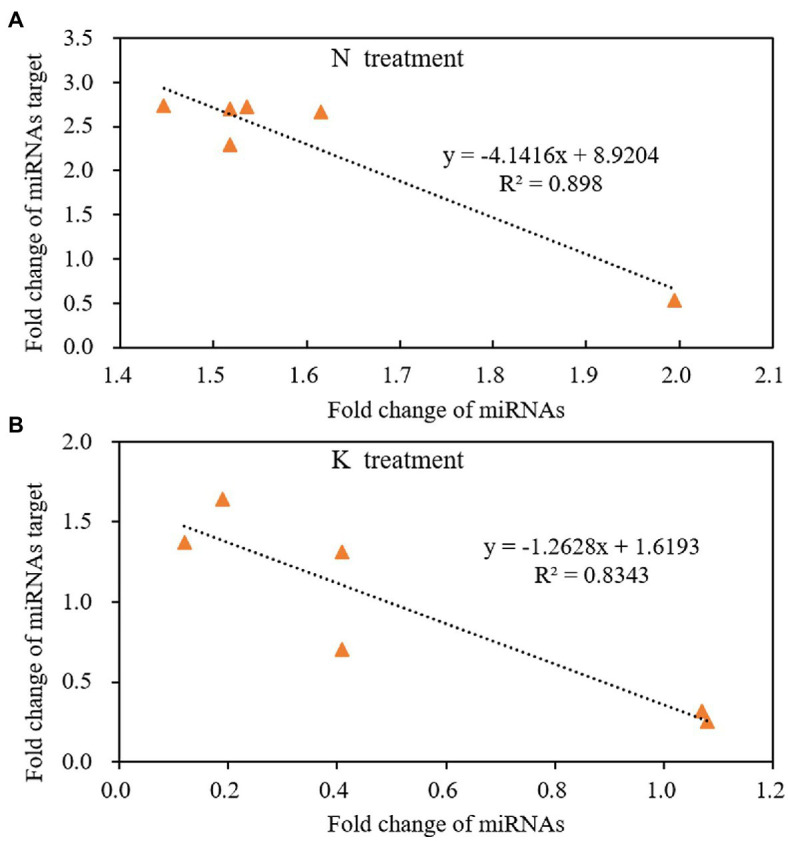
The reverse liner relationship of fold change between miRNAs and their targets under N treatment **(A)** and K treatment **(B)** in the root of peanut seedlings. N, nitrogen and K, potassium.

miRNA regulates gene expression by shearing target mRNAs or inhibiting protein translation. Therefore, the expression of miRNA is negatively correlated with the expression of its targeted mRNAs. That is, if miRNA is upregulated, the expression of its target gene should be downregulated. The expression pattern of miRNAs and their target mRNA are often complex when secondary reactions and other gene regulatory factors besides miRNAs. In this case, the reverse expression relationship between the miRNAs and their target gene may not be observed. In the present study, we used linear equations to simulate the relationship between the fold change of miRNAs and their targeted mRNAs from the 4th day of treatment to the 8th day of treatment. The results showed that the expression of miRNAs had a negative linear relationship with their targets under both N deficiency and K deficiency treatment. And the reverse relationship could be described by the equation: *y* = −4.1416x + 8.9204 (*R*^2^ = 0.898, Figure 7A) and the equation: *y* = −1.2628x + 1.6193 (*R*^2^ = 0.8343, Figure 7B) under N deficiency and K deficiency treatments, respectively. It indicates that the tested miRNAs negatively regulate the expression of their targeted mRNAs.

## Discussion

### N and K Deficiency Significantly Inhibited the Growth and Development of Peanut Seedlings

Nitrogen is the main component of many important compounds in plants. It participates in plant photosynthesis, a series of biochemical reactions, and plays an important role in biomass accumulation and yield formation in crops (Hawkesford et al., 2012; Kant, 2018). Potassium is the quality element of plants, which participates in osmotic adjustment, photosynthesis, material transport, and other processes, and it can improve the stress resistance of plants (Helmy and Ramadan, 2014; Almeida et al., 2015; Chakraborty et al., 2016). Therefore, the deficiency of these two kinds of macro-elements will lead to the inhibition of plant growth. In this study, the dry weight of shoot and root, leaf area, and plant height of peanut seedlings were significantly reduced under both 0.1 mM NO_3_^−^ and 0.02 mM K^+^ treatment. It meant that the 0.1 mM NO_3_^−^ and 0.02 mM K^+^ caused the low N stress and low K stress to the peanut seedlings, respectively, and inhibited the growth of peanut seedlings. These N deficiency-induced growth inhibition phenomena were also observed in other plant species, including potatoes (Xie et al., 2018). Studies have also shown that 0.02 mM K^+^ can induce aberrant growth and development in cotton (Fontana et al., 2020) and wheat (Thornburg et al., 2020), which probably due to the reduction of chlorophyll content and photosynthetic capacity.

### The Peanut Root System Has a Specific Response to N Deficiency and K Deficiency Stresses

Roots are the main organ for plants to absorb nutrients and water. It is also the first organ to sense the changes of rhizosphere environmental conditions and shows strong plasticity to adapt to the external environment changes (Malamy, 2005). Analysis of RSA provides good quantitative readouts that can be used to identify the genes and signaling pathways, enabling plants to sense changes in the root environment and integrate them into adaptive responses (Kellermeier et al., 2014). The traditional view is that each nutrient deficiency will lead to a typical root structure (Kellermeier et al., 2014). This study also found that the peanut root system has a different response to N deficiency and K deficiency conditions. Previous studies have shown that plants intend to develop exploratory roots with more lateral roots (Araya et al., 2014; Kant, 2018). Mohd-Radzman and coworkers demonstrated that a uniform low concentration of nitrate (e.g., ≤1 mM) enhanced the growth of both lateral and primary roots of legumes (Mohd-Radzman et al., 2013). While our result about the peanut root system has a specific response to low N stress, both the lateral and primary root growth was inhibited under N deficiency conditions. Studies in *Arabidopsis* showed that under mild N deficiency, the average length of the lateral root was significantly promoted, while under severe N deficiency, the total lateral root length decreased, and there was almost no lateral root formation (Mohd-Radzman et al., 2013). Forde (2014) considered that the effect of low N stress or N deficiency on root branches depends on the N nutrition status of plants and the degree of stress on plants. Based on the above results, it can be considered that different crop roots respond differently to N deficiency, and root growth is related to the degree of N deficiency stress.

Previous studies have demonstrated that K deficiency has a negative effect on root elongation and the number of first-order lateral roots (Jung et al., 2009; Kellermeier and Amtmann, 2013), but the response varies among ecotypes, cultivars, species, and even root types (Kellermeier and Amtmann, 2013; Sustr et al., 2019). In this study, low K promotes both lateral and primary root growth while reduced the total root length. Similar results were also observed in wheat (Thornburg et al., 2020), and almost the opposite result was observed in cotton (Fontana et al., 2020). Although the number of lateral roots and the primary root length increased, the root system was inhibited under K deficiency treatment. The inhibition of root growth may be related to K deficiency induced slows down cell volume growth or restricts the transport of assimilation from the phloem to underground organs (Cakmak et al., 1994a,b).

Root vitality can represent the absorption, synthesis, oxidation, and reduction capacity of the root system and is a comprehensive index reflecting the absorption function of roots. 2,3,5-triphenyltetrazolium chloride is often used as a quantitative indicator of cell activity and is a good root metabolic reactive agent (Cakmak et al., 1994a,b). TTC can receive electrons directly from the electron transport link, and its reduction is directly related to mitochondrial respiration rate (Comas et al., 2000; Ruf and Brunner, 2003). Therefore, root vitality is the use of dehydrogenase activity in the respiratory chain to reflect the physiological indicators of root life activities. It is also a reflection of the strength of respiration. In this study, N deficiency and K deficiency significantly decreased the root vitality and the O_2_ consuming rate, representing the root respiration rate. Fontana et al. (2020) also found that K deficiency can cause the decline of root vitality and root respiration rate in cotton seedling planted in a low K solution that contained KCl (0.02 mM) for 8 days. The study in wheat showed that low N stress weakens the key enzyme activities of the NADP dehydrogenase system in roots. The decrease of NADPH synthesis affects the respiratory and metabolic activities of roots (Zhou et al., 2018). These results showed that N deficiency and K deficiency blocked root metabolism, decreased absorption capacity, and inhibited root growth, resulting in decreased root vitality.

### miRNA Response to N and K Deficiency in Plants

N and K deficiency altered the expression of certain miRNAs and miRNA-mediated genes. There were differences in the miRNA expression patterns between the plants cultivated in N deficiency nutrient solution and those cultivated in K deficiency nutrient solution. Nguyen et al. (2015) observed that under N deficiency stress, the expression of miR156, miR160, and miR171 was induced, and the expression of miR169, miR172, miR319, miR396, and miR399 were reduced in *Arabidopsis* roots, while the expression of these miRNAs in maize roots was opposite. Therefore, it can be seen that the miRNA expression patterns varied with crop varieties and N deficiency. In this study, all 20 tested miRNAs, except for miR160, miR164, miR171, miR393, and miR827, were upregulated after 4 and 8 days of K deficiency treatments (Figure 5B). These may be the molecular mechanism of the adaptive response of peanut roots to N and K deficiency.

Previous studies have shown that miRNAs regulate genes encoding AUXIN RESPONSE FACTORS (ARFs) after transcription (Williams et al., 2005; Yuan et al., 2019). ARFs can specifically combine with auxin response elements in the auxin response gene promoter region to activate or inhibit gene expression, thus regulating plant growth and development (Hagen and Guilfoyle, 2002). Conserved miR160 regulates root growth and gravitational properties by negatively regulating three ARFs (ARF10, 16, and 17). In plants, overexpressing mir160c, ARF10, and ARF16 mRNA expression was decreased. The roots were short and attractive, with tumor-like apex related to uncontrolled cell division of apical meristem and failure of small columnar cell differentiation (Wang et al., 2005). The conserved miR167 negatively regulates auxin signaling by targeting ARF6 and ARF8. Under the condition of high nitrate, the expression level of miR167 in the root of *Arabidopsis* was decreased, allowing the accumulation of ARF8 in the pericyclic cell and lateral root cap, thereby stimulating the auxin signal, promoting lateral root formation, but inhibiting root elongation (Gifford et al., 2008). Auxin responsive miR390 is specifically expressed in the basal part of lateral root primordia and the potential vascular parenchymal cells and act as a regulatory factor by triggering the biogenesis of TAS3-derived transacting short-interfering RNAs (tasiRNAs; Marin et al., 2010). These tasiRNAs can repress the expression of ARF2, ARF3, and ARF4, thus promoting the development progression of lateral root (Marin et al., 2010; Du and Scheres, 2017). In this study, miR160, miR167, and miR390 downregulated on the 4th day and upregulated on the 8th day of N deficiency treatment. While ARF upregulated on the 4th day and downregulated on the 8th day of N deficiency treatment. miR160 upregulated on the 4th day and downregulated on the 8th day of K deficiency treatment. miR167 was nearly 6.32- and 1.67-fold upregulated on the 4th and 8th days of K deficiency treatment, respectively. The expression of miR167 on the 8th day was significantly lower than that on the 4th day of K deficiency. In addition, miR390 was upregulated on both the 4th and 8th days of K deficiency, and the ARF2 expression increased on both the 4th and 8th days of K deficiency. Thus, the altered expression of miR160, miR167, and miR390/ARF2 may be related to the inhibition of lateral root under N deficiency and the enhancement of lateral root under K deficiency treatment.

In addition to ARF-mediated auxin signal transduction, miRNAs also regulated auxin perception (Yuan et al., 2019). Studies have demonstrated that conserved miR393 targets genes encoding a small number of F-box-containing auxin receptors in many plants, including TRANSPORT INHIBITOR RESPONSE1 (TIR1) and AUXIN SIGNALING F-BOX (AFBs; Yuan et al., 2019), which are important for the growth of both primary root and lateral root (Vidal et al., 2013). miR393/AFB3 has been considered a nitrate responsive regulatory module that can integrate N and auxin signaling to regulate root growth in plant response to nitrate availability (Vidal et al., 2010, 2013). In this study, miR393 was downregulated on the 4th day and upregulated on the 8th day of N deficiency treatment. The expression of AFB3 was upregulated on the 4th day and further upregulated on the 8th day of N deficiency treatment. The expression pattern of miR393 under K deficiency stress is the opposite of its expression pattern under N deficiency stress. The expression pattern of miR393 was upregulated on the 4th day and downregulated on the 8th day of K deficiency treatment. The expression pattern of AFB3 was the opposite of the expression pattern of miR393 under K deficiency stress. The different changes of miR393/AFB3 under N deficiency and K deficiency conditions may explain the different responses of lateral root number and root length under these two conditions.

miR396 is a conserved miRNA in plants, closely related to plant growth and development (Debernardi et al., 2012; Li and Zhang, 2016). It has been found that miR396 targets the GROWTH REGULATION FACTORS (GRF) gene family and bHLH74, which promote cell proliferation during leaf development (Debernardi et al., 2012). miR396 may regulate root growth by restricting cell proliferation in root apical meristems (Debernardi et al., 2012). In this study, miR396 was significantly upregulated, and its target GRF was significantly downregulated on the 8th day of N deficiency. Thus, the altered expression of miR396/GRF may be related to the inhibition of lateral root under N deficiency treatment. Under K deficiency, miR396 was significantly upregulated on both the 4th and 8th days and compared with the expression on the 4th day, the expression of miR396 on the 8th day was significantly decreased. In contrast, the expression of GRF was significantly upregulated on both the 4th and 8th days of K deficiency. Therefore, the upregulation of GRF could be the reason for the enhancement of root growth in peanut under K deficiency.

Genes of the NAC (NAMATAF-CUC) family are the auxin signal transduction genes responsible for lateral root emergence and have been identified as the target of miR164 (Guo et al., 2005). Plants with low miR164 expression have high NAC1 expression and produced more lateral roots (Guo et al., 2005). In this study, miR164 was downregulated on the 4th day and upregulated on the 8th day of N deficiency. While the expression pattern of miR164 under K deficiency stress is just the opposite of its expression pattern under N deficiency stress. The expression of NAC was upregulated on the 4th day and downregulated on the 8th day of K deficiency. Because NAC transduces auxin signal for lateral root emergence (Guo et al., 2005), the upregulation of NAC under potassium deficiency further indicated that potassium deficiency induced the emergence of lateral roots.

In plants, miR156 targets some SPL (squamosa promoter binding protein-like) genes, which are plant-specific transcription factors in many developmental processes (Gou et al., 2011; Yu et al., 2015). In *Arabidopsis*, 17 members of the *SPL* gene family, 11 of which are regulated by miR156 (Qian et al., 2017). Yu et al. (2015) found that miR156 was differentially expressed in specific cells and tissues of lateral roots and responded to auxin signal transduction pathway by regulating *SPL3*, *SPL9*, and *SPL10* genes, thereby inhibiting the growth of lateral roots, in which SPL10 played a leading role. In the present study, miR156 was nearly 4.3- and 4.6-fold induced on 4th and 8th days of K deficiency treatment. The SPL10 gene was 1.54-fold upregulated on the 4th day and was 0.49-fold downregulated on the 8th day of K deficiency treatment. The upregulated miR156 and the downregulated SPL10 may be the reason for initiating lateral roots under the K deficiency condition. Additionally, the upregulated SPL10 may be the reason for the lower number of lateral roots under the N deficiency condition. Plants overexpressing miR156 produced more lateral roots, while plants with lower miR156 levels had fewer lateral roots (Yu et al., 2015). SPLs can also directly activate miR172 and induce the floral inducer FLOWERING LOCUS (FT) through miR172-mediated AP2 inhibition, promoting plant flowering (Song et al., 2019). Over-expression of miR172 confers early flowering (Wang et al., 2016) and increased nodule numbers in soybean (Wang et al., 2014). A study in *Arabidopsis* suggested that miR172 acts downstream of miR156 and mediated the effect of miR156 on flowering time and the change of vegetative phase (Wu et al., 2009). Further study of the functions of these miRNAs and their regulated gene network will elucidate the molecular mechanism of peanut response to nutrient deficiency, which provides a new strategy for peanut molecular improvement using advanced technology, including the CRISPR/Cas genome editing (Li et al., 2021; Zhang et al., 2021).

## Conclusion

**Figure 8:**
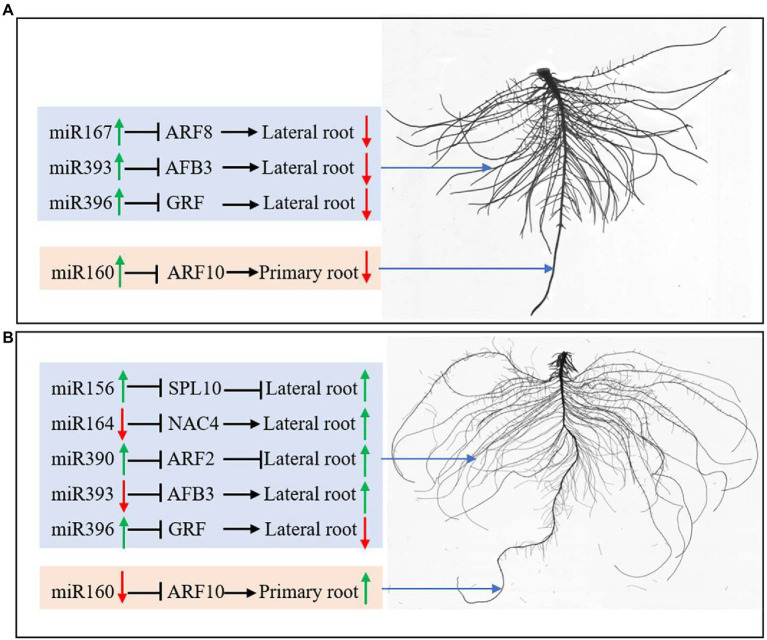
miRNAs regulate root development by controlling protein-coding genes’ expression under N deficiency **(A)** and K deficiency **(B)**. The green arrow represents the upregulated expression of miRNAs and the enhanced growth of roots; the red arrow represents the downregulated expression of miRNAs and the inhibited growth of roots. N, nitrogen and K, potassium.

In this study, both N deficiency and K deficiency caused abnormal growth and development of peanut seedlings. They changed the expression of miRNAs and their target genes related to root development. Under N deficiency condition, the lateral root initiation and emergence inhibition may be related to the upregulation of miR164, miR393, and miR396 and the downregulated AFB3 and GRF (Figure 8A). The inhibition of primary root elongation may be associated with the upregulation of miR160 under N deficiency condition. Under the K deficiency condition, the upregulated miR156, miR390, NAC4, ARF2, and AFB3, and the downregulated miR164, miR393, and SPL10 may be involved in the enhancement in the lateral root initiation and emergence (Figure 8B). Downregulated miR160 probably contributes to promoting primary root elongation. Overall, the roots of a peanut had different responses to N deficiency and K deficiency stresses, which is potentially due to the miRNAs-mediated pathway and mechanism. In conclusion, the different responses of peanut roots to nitrogen and potassium deficiency stress may be related to miRNA-mediated regulation of root growth and development.

## Data Availability Statement

The original contributions presented in the study are included in the article/supplementary material, further inquiries can be directed to the corresponding authors.

## Author Contributions

LL: data curation, formal analysis, investigation, writing – original draft, and writing – review and editing. QL and WL: data curation and investigation. KD: methodology and investigation. CP: methodology and software. SO: investigation and validation. JL and GW: methodology, data curation, and investigation. JF and TT: investigation. IP: investigation and revision of the manuscript. FL: investigation and software. ZZ: conceptualization, methodology, resources, supervision, funding acquisition, and writing – original draft. YZ: conceptualization, methodology, resources, and supervision. XP: conceptualization, methodology, supervision, and funding acquisition. BZ: conceptualization, methodology, resources, supervision, and funding acquisition. All authors contributed to the article and approved the submitted version.

### Conflict of Interest

The authors declare that the research was conducted in the absence of any commercial or financial relationships that could be construed as a potential conflict of interest.
